# Supplementary material for: Macrophage scavenger receptor 1 controls Chikungunya virus infection through autophagy in mice
Source: Commun Biol. 2020 Oct 8;3:556. doi: 10.1038/s42003-020-01285-6 (PMC7545163; doi:10.1038/s42003-020-01285-6)
Supplement: Supplementary file 1 — Supplementary Information [file 42003_2020_1285_MOESM1_ESM.pdf]

Macrophage scavenger receptor 1 controls Chikungunya virus infection through autophagy in mice

Long Yang, Tingting Geng, Guang Yang, Jinzhu Ma, Leilei Wang, Harshada Ketkhar, Duomeng Yang, Tao Lin, Jesse Hwang, Shu Zhu, Yanlin Wang, Jianfeng Dai, Fuping You, Gong Cheng, Antony Vella, Richard. A Flavell, Erol Fikrig, Penghua Wang

This Supplementary Information contains Supplementary Figs.1- 3 and Supplementary Table 1.

Supplementary Fig.1

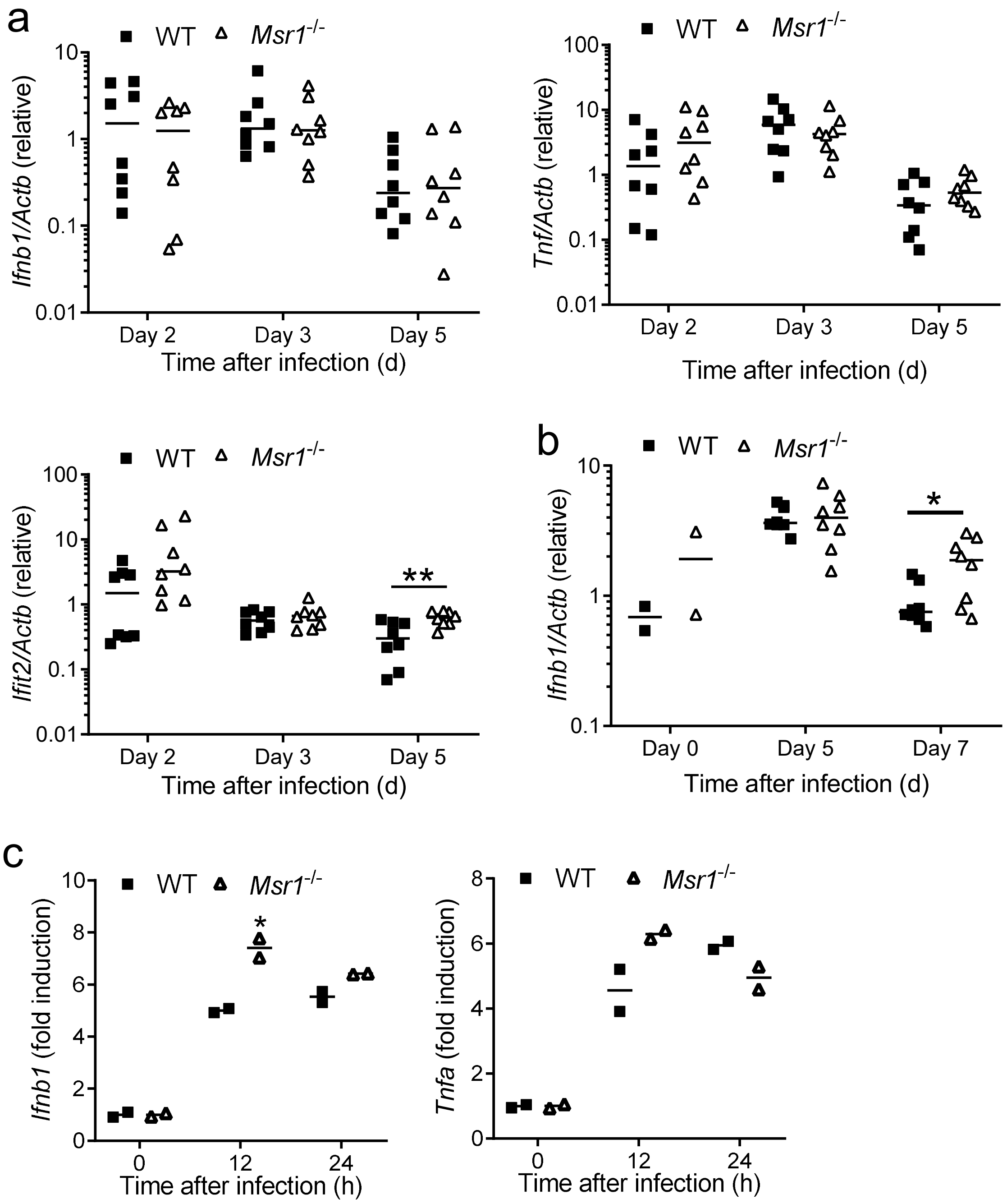

Supplementary Fig.1 MSR1 is dispensable for CHIKV-induced type I IFN response. a, b) 8 weeks old, sex-matched, C57BL/6 (WT) and Msr1 deficient (*Msr1*$^{-/-}$) mice were infected with CHIKV. Quantitative RT-PCR analyses of selected immune gene expression in **a**) whole blood cells and **b**) the ankle joints. *, P<0.05, **, P<0.01 (non-parametric Mann-Whitney U test). **c**) Quantitative RT-PCR analyses of the select immune genes in primary bone marrow-derived macrophages infected with CHIKV at a multiplicity of infection (MOI) of 10. N=2. *, P<0.05 (two-way ANOVA). Each dot represents one mouse/biological repeat; the small horizontal line indicates the median of the result. The data are representative of two independent experiments.

Supplementary Fig.2

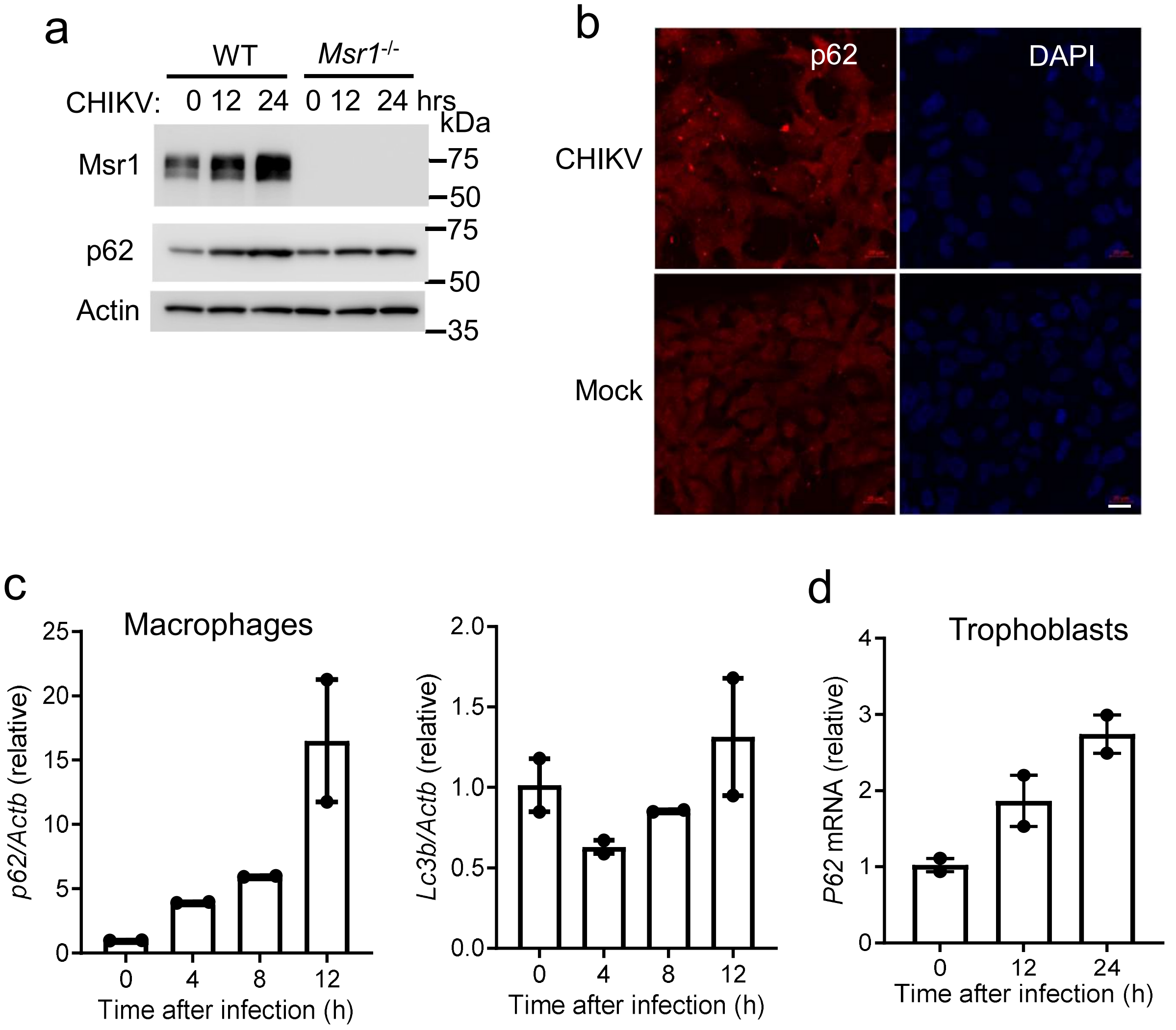

Supplementary Fig.2 P62 expression is inducible by CHIKV. **a**) Immunoblots showing p62 levels in primary bone marrow derived mouse macrophages (BMDM) at various times after CHIKV at a multiplicity of infection (MOI) of 10. **b**) Microscopic images of immunofluorescent staining for P62 in trophoblasts without (mock) /with CHIKV at a MOI of 0.5 for 12hrs. P62 was stained by a rabbit antibody, followed by an Alexa Fluor-594 (red) conjugated secondary antibody. The cells were counterstained for nuclei by DAPI (blue). The images were acquired using a Zeiss LSM 880 confocal microscope (400x). Scale bar: 20μM. Quantitative RT-PCR analyses of **c**) *p62* and *Lc3b* mRNA in BMDMs infected with CHIKV (MOI=10), **d**) *P62* mRNA in human trophoblasts infected with CHIKV (MOI=0.5). Bars: mean + s.e.m, n=2.

Supplementary Fig.3

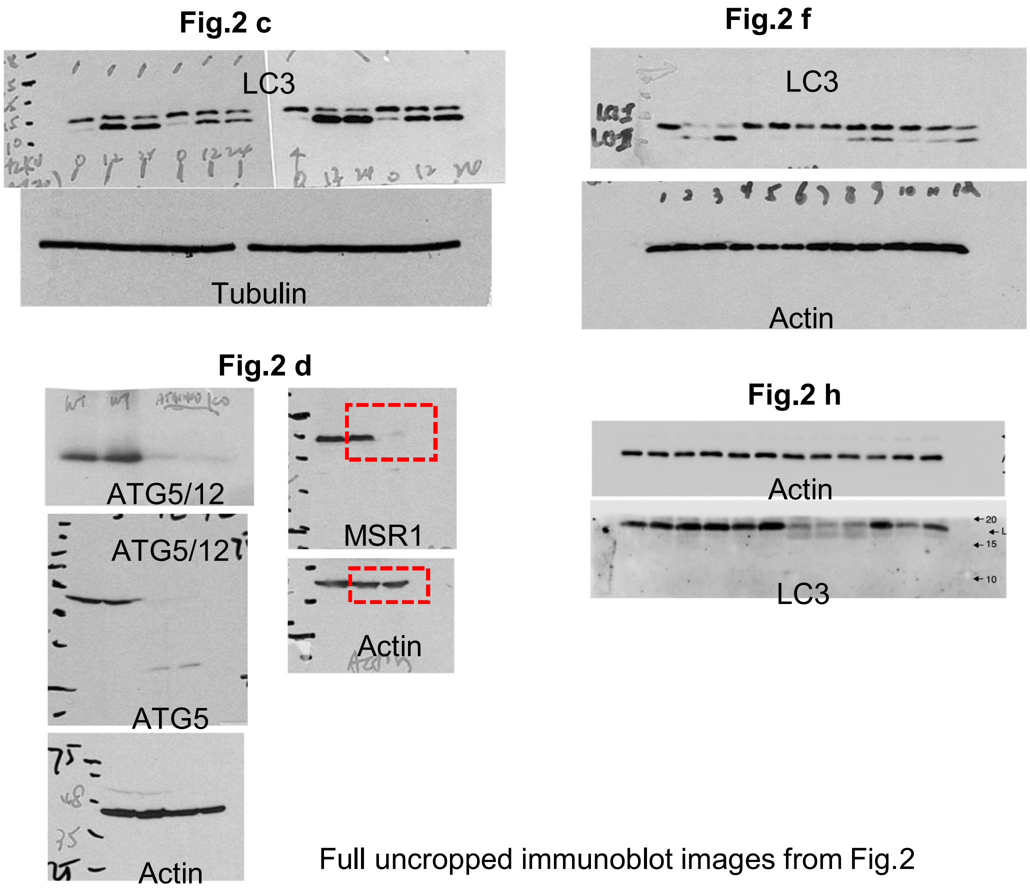

Full uncropped immunoblot images from Fig.2

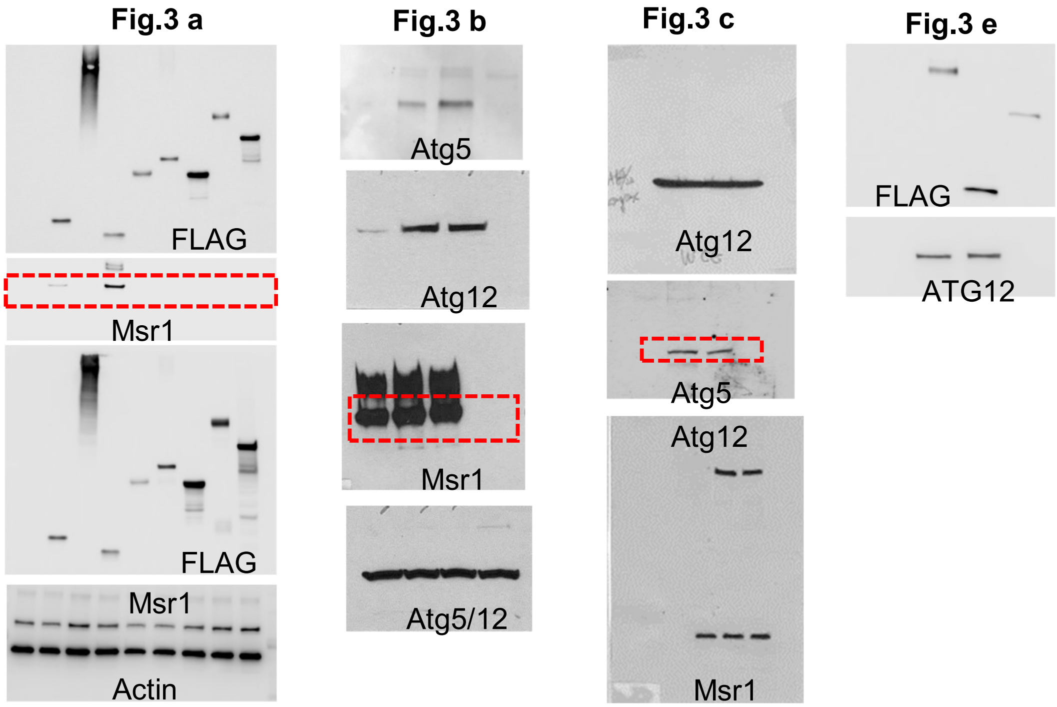

Full uncropped immunoblot images from Fig.3

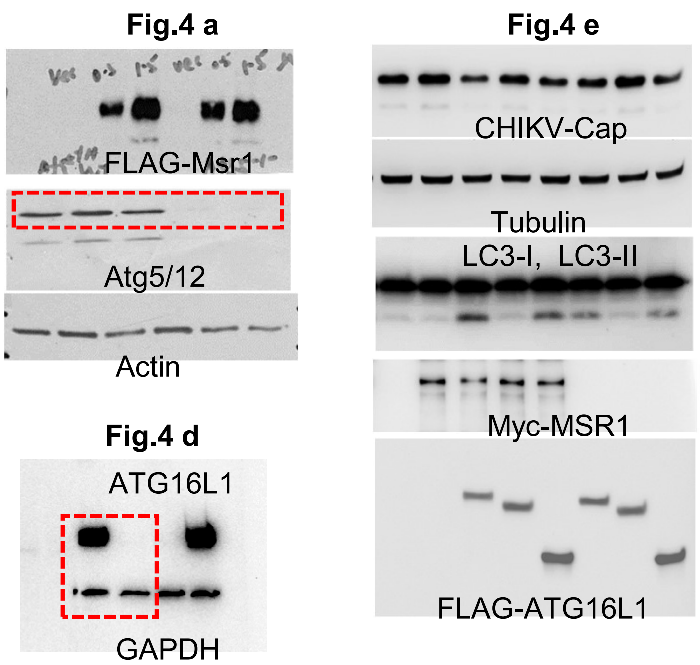

Full uncropped immunoblot images from Fig.4

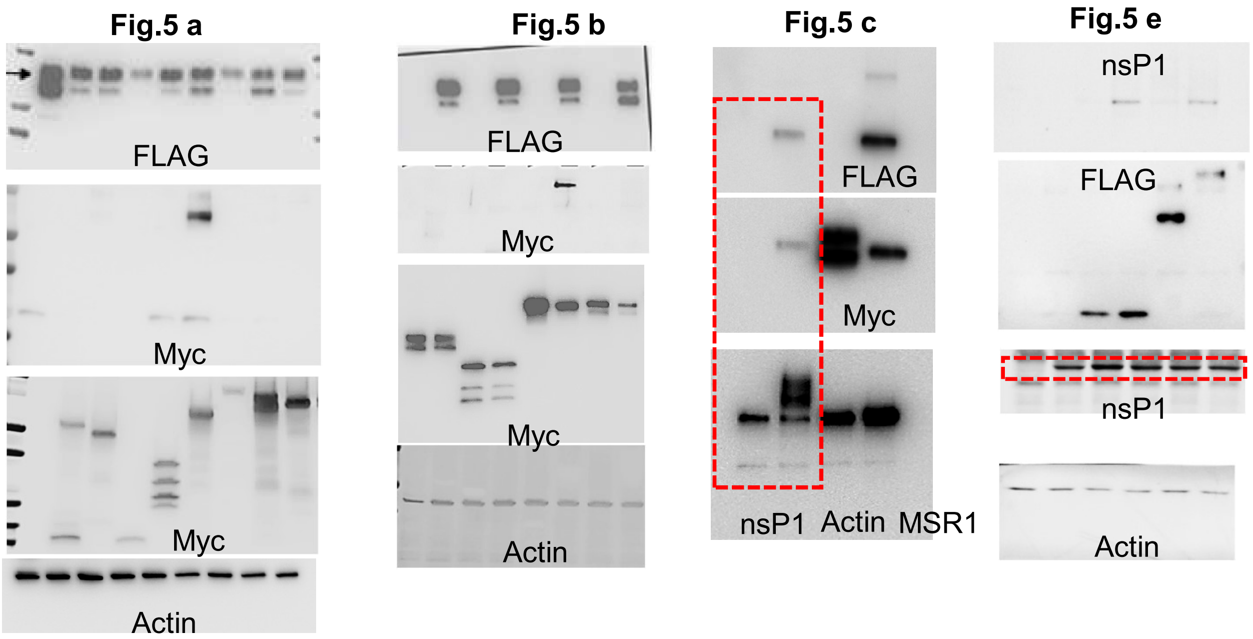

Full uncropped immunoblot images from Fig.5

Fig.6 a

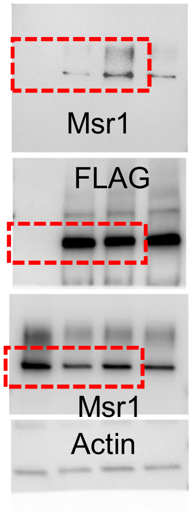

Fig.6 b

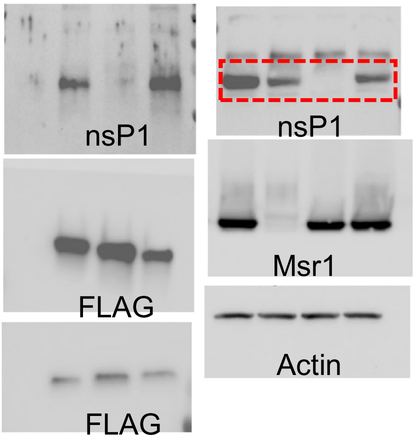

Fig.6 c

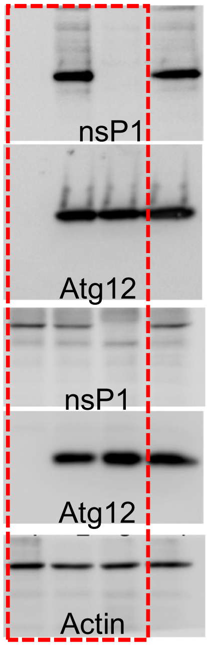

Fig.6 e

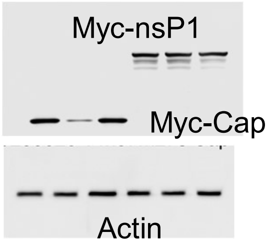

Fig.6 f

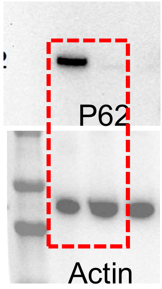

Fig.6 g

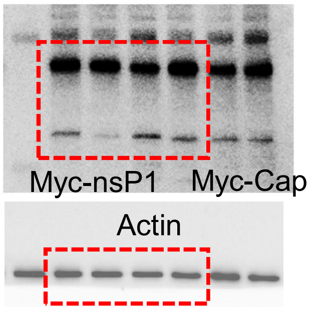

Full uncropped immunoblot images from Fig.6

Supplementary Fig.2 a

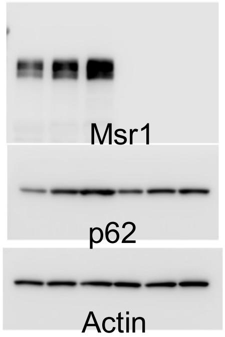

Full uncropped immunoblot images from Supplementary Fig.2

Supplementary Fig.3 Full uncropped immunoblot images from main and supplementary figures.

Supplementary Table 1

Gene symbol	Species	Forward primer (5'-3')	Reverse primer (5'-3')	Remark
**Primers for qPCR**				
MSR1	Hs	TGCACAAGGCAGCTCACTTTGG	GTGCAAGTGACTCCAGCATCTTC	
Msr1	Mm	AGTGCTGTCTTCTTTACCAGC	GTGAGGAAGGGATGCTGTA	
nsP2	CHIKV	GGCAGTGGTCCCAGATAATTCAAG	GCTGTCTAGATCCACCCCATACATG	
Lc3b	Mm	GTCCTGGACAAGACCAAGTTCC	CCATTCACCAGGAGGAAGAAGG	
Sqstm1/p62	Mm	GCTCTTCGGAAGTCAGCAAACC	GCAGTTTCCCGACTCCATCTGT	
LC3B	Hs	GAGAAGCAGCTTCCTGTTCTGG	GTGTCCGTTCACCAACAGGAAG	
SQSTM1/p62	Hs	TGTGTAGCGTCTGCGAGGGAAA	AGTGTCCGTGTTTCACCTTCCG	
Tnfa	Mm	CTCCAGGCGGTGCCTATGT	GAAGAGCGTGGTGGCCC	Probe: CAGCCTCTTCTCATTCCTGCTTGTGGC
Ifna	Mm	CTTCCACAGGATCACTGTGTACCT	TTCTGCTCTGACCACCTCCC	Probe: AGAGAGAAGAAACACAGCCCCTGTGCC
Ifnb1	Mm	CTGGAGCAGCTGAATGGAAAG	CTTCTCCGTCATCTCCATAGGG	Probe: CAACCTCACCTACAGGGCGGACTTCAAG
Actb	Mm	AGAGGGAAATCGTGCGTGAC	CAATAGTGATGATGACCTGGCCGT	Probe: CACTGCCGCATCCTCTTCCTCCC
ACTB	Hs	ATCCTGGCCTCGCTGTCCAC	GGGCCGGACTCGTCATAC	
**Guide RNA for CRISPR-Cas9 knockout**				
ATG16L1	Hs	GGGGGAAGTCAGCGGCGCGG		
ATG12	Hs	GTCTTCCGCTGCAGTTTCCC		
MSR1	Hs	TGATCTCAGACTGAAAGATT		
SQSTM1/P62	Hs	CGAGCCTGAGGCGGAAGCCG		

Supplementary Table 1 Oligo DNA sequences. Homo sapiens (Hs), Mus musculus (Mm), Chikungunya virus (CHIKV).
