# Supplementary material for: Macrophage scavenger receptor 1 controls Chikungunya virus infection through autophagy in mice
Source: Commun Biol. 2020 Oct 8;3:556. doi: 10.1038/s42003-020-01285-6 (PMC7545163; doi:10.1038/s42003-020-01285-6)
Supplement: Supplementary file 4 — Reporting Summary [file 42003_2020_1285_MOESM4_ESM.pdf]

nature research

Corresponding author(s): Penghua Wang

Last updated by author(s): 09/07/2020

Reporting Summary

Nature Research wishes to improve the reproducibility of the work that we publish. This form provides structure for consistency and transparency in reporting. For further information on Nature Research policies, see our Editorial Policies and the Editorial Policy Checklist.

Statistics

For all statistical analyses, confirm that the following items are present in the figure legend, table legend, main text, or Methods section.

n/a	Confirmed	
☐	☒	The exact sample size (n) for each experimental group/condition, given as a discrete number and unit of measurement
☐	☒	A statement on whether measurements were taken from distinct samples or whether the same sample was measured repeatedly
☐	☒	The statistical test(s) used AND whether they are one- or two-sided<br>*Only common tests should be described solely by name; describe more complex techniques in the Methods section.*
☐	☒	A description of all covariates tested
☒	☐	A description of any assumptions or corrections, such as tests of normality and adjustment for multiple comparisons
☐	☒	A full description of the statistical parameters including central tendency (e.g. means) or other basic estimates (e.g. regression coefficient) AND variation (e.g. standard deviation) or associated estimates of uncertainty (e.g. confidence intervals)
☒	☐	For null hypothesis testing, the test statistic (e.g. F, t, r) with confidence intervals, effect sizes, degrees of freedom and P value noted<br>*Give P values as exact values whenever suitable.*
☒	☐	For Bayesian analysis, information on the choice of priors and Markov chain Monte Carlo settings
☒	☐	For hierarchical and complex designs, identification of the appropriate level for tests and full reporting of outcomes
☒	☐	Estimates of effect sizes (e.g. Cohen's d, Pearson's r), indicating how they were calculated

Our web collection on statistics for biologists contains articles on many of the points above.

Software and code

Policy information about availability of computer code

Data collection	N/A
Data analysis	N/A

For manuscripts utilizing custom algorithms or software that are central to the research but not yet described in published literature, software must be made available to editors and reviewers. We strongly encourage code deposition in a community repository (e.g. GitHub). See the Nature Research guidelines for submitting code & software for further information.

Data

Policy information about availability of data

All manuscripts must include a data availability statement. This statement should provide the following information, where applicable:

- Accession codes, unique identifiers, or web links for publicly available datasets
- A list of figures that have associated raw data
- A description of any restrictions on data availability

Fig.1, 2, 4 have associated source data. The datasets generated during and/or analysed during the current study are available from the corresponding author on reasonable request.

Field-specific reporting

Please select the one below that is the best fit for your research. If you are not sure, read the appropriate sections before making your selection.

☒ Life sciences ☐ Behavioural & social sciences ☐ Ecological, evolutionary & environmental sciences

For a reference copy of the document with all sections, see nature.com/documents/nr-reporting-summary-flat.pdf

Life sciences study design

All studies must disclose on these points even when the disclosure is negative.

Sample size	The sample size chosen for our animal experiments in this study was estimated based on our prior experience of performing similar sets of experiments and power analysis calculations (http://isogenic.info/html/power_analysis.html).
Data exclusions	All animal results were included.
Replication	2-3 independent reproducible experiments were performed. At least 2 biological replicates were included for each experiment/each condition where a statistical analysis was performed.
Randomization	All animal results were included and no method of randomization was applied.
Blinding	H&E slides were scored in a blind manner

Reporting for specific materials, systems and methods

We require information from authors about some types of materials, experimental systems and methods used in many studies. Here, indicate whether each material, system or method listed is relevant to your study. If you are not sure if a list item applies to your research, read the appropriate section before selecting a response.

Materials & experimental systems

n/a	Involved in the study	
☐	☒	Antibodies
☐	☒	Eukaryotic cell lines
☒	☐	Palaeontology and archaeology
☐	☒	Animals and other organisms
☒	☐	Human research participants
☒	☐	Clinical data
☒	☐	Dual use research of concern

Methods

n/a	Involved in the study	
☒	☐	ChIP-seq
☒	☐	Flow cytometry
☒	☐	MRI-based neuroimaging

Antibodies

Antibodies used	The rabbit anti LC3B (Cat # 2775), GAPDH (Cat #5174), Tubulin (Cat# 2148), ATG5 (Cat#12994), Actin (Cat # 8456) and anti-p62/SQSTM11(Cat# 5114, #7695) antibodies were purchased from Cell Signaling Technology (Danvers, MA 01923, USA). The mouse anti-FLAG (Cat# TA50011) and rabbit anti-human/mouse MSR1 (Cat# TA336699) antibodies were from Origene (Rockville, MD 20850, USA); the goat anti-mouse MSR1 (Cat# AF1797), mouse ant-human MSR1 (Cat# MAB2708), mouse anti-ATG12 (Cat# MAB6807) from R&D Systems (Minneapolis, MN 55413, USA). The mouse anti-CHIKV (Clone A54Q, Cat# MA5-18181) was obtained from ThermoFisher Scientific (Rockford, IL 61105, USA). The rat anti-CHIKV nsP1 (Cat# 111441) and nsP2 (Cat# 111442) were available from Antibody Research Corporation (St Peters, MO 63304, USA) and rabbit anti-CHIKV nsP1 (Cat# 11-13020) from ABGENEX (Bhubaneswar, Odisha 751024, India).
Validation	These antibodies have been validated by published studies elsewhere. We also validated some of them with specific gene knockout cell lysates or recombinant proteins.

Eukaryotic cell lines

Policy information about cell lines

Cell line source(s)	Human embryonic kidney 293 cells transformed with T antigen of SV40 (HEK293T, # CRL-3216), Vero cells (monkey kidney epithelial cells, # CCL-81), immortalized human trophoblasts HTR-8/SVneo (#CRL-3271) and L929 (mouse fibroblast cells, # CCL-1) were purchased from American Type Culture Collection (ATCC) (Manassas, VA20110, USA).
Authentication	These cell lines are from and have been authenticated by ATCC.

Mycoplasma contamination	In order to ensure the cell culture free of mycoplasma, we regularly treated cells with MycoZap (Lonza).
Commonly misidentified lines (See ICLAC register)	These cell lines are not listed in the database of commonly misidentified cell lines maintained by ICLAC.

Animals and other organisms

Policy information about studies involving animals; ARRIVE guidelines recommended for reporting animal research

Laboratory animals	All mice were obtained from the Jackson Laboratory and had the same genetic background (C57BL/6) and housing conditions. The control mice were C57BL/6. Msr1-/- mice were made from 129 embryonic stem (ES) cells and then backcrossed to C57BL/6 mice for 12 generations (https://www.jax.org/strain/006096).
Wild animals	N/A
Field-collected samples	N/A
Ethics oversight	All animal protocols were approved by the Institutional Animal Care & Use Committee at Yale University, New York Medical College and UConn Health adhering to the National institutes of Health recommendations for the care and use of laboratory animals.

Note that full information on the approval of the study protocol must also be provided in the manuscript.
